# Progress and opportunities of foundation models in bioinformatics

**DOI:** 10.1093/bib/bbae548

**Published:** 2024-10-26

**Authors:** Qing Li, Zhihang Hu, Yixuan Wang, Lei Li, Yimin Fan, Irwin King, Gengjie Jia, Sheng Wang, Le Song, Yu Li

**Affiliations:** Department of Computer Science and Engineering, The Chinese University of Hong Kong, Shatin, New Territories, Hong Kong SAR, 999077, China; Department of Computer Science and Engineering, The Chinese University of Hong Kong, Shatin, New Territories, Hong Kong SAR, 999077, China; Department of Computer Science and Engineering, The Chinese University of Hong Kong, Shatin, New Territories, Hong Kong SAR, 999077, China; Department of Computer Science and Engineering, The Chinese University of Hong Kong, Shatin, New Territories, Hong Kong SAR, 999077, China; Department of Computer Science and Engineering, The Chinese University of Hong Kong, Shatin, New Territories, Hong Kong SAR, 999077, China; Department of Computer Science and Engineering, The Chinese University of Hong Kong, Shatin, New Territories, Hong Kong SAR, 999077, China; Shenzhen Branch, Guangdong Laboratory of Lingnan Modern Agriculture, Genome Analysis Laboratory of the Ministry of Agriculture and Rural Affairs, Agricultural Genomics Institute at Shenzhen, Chinese Academy of Agricultural Sciences, Shenzhen, Guangdong, 518120, China; Shanghai Zelixir Biotech Company Ltd., Shanghai, 200030, China; Shenzhen Institute of Advanced Technology, Xueyuan Avenue, Shenzhen University Town, Nanshan District, Shenzhen, Guangdong, 518055, China; BioMap, Zhongguancun Life Science Park, Haidian District, Beijing, 100085, China; Department of Computer Science and Engineering, The Chinese University of Hong Kong, Shatin, New Territories, Hong Kong SAR, 999077, China

**Keywords:** foundation models, large language models, bioinformatics, artificial intelligence

## Abstract

Bioinformatics has undergone a paradigm shift in artificial intelligence (AI), particularly through foundation models (FMs), which address longstanding challenges in bioinformatics such as limited annotated data and data noise. These AI techniques have demonstrated remarkable efficacy across various downstream validation tasks, effectively representing diverse biological entities and heralding a new era in computational biology. The primary goal of this survey is to conduct a general investigation and summary of FMs in bioinformatics, tracing their evolutionary trajectory, current research landscape, and methodological frameworks. Our primary focus is on elucidating the application of FMs to specific biological problems, offering insights to guide the research community in choosing appropriate FMs for tasks like sequence analysis, structure prediction, and function annotation. Each section delves into the intricacies of the targeted challenges, contrasting the architectures and advancements of FMs with conventional methods and showcasing their utility across different biological domains. Further, this review scrutinizes the hurdles and constraints encountered by FMs in biology, including issues of data noise, model interpretability, and potential biases. This analysis provides a theoretical groundwork for understanding the circumstances under which certain FMs may exhibit suboptimal performance. Lastly, we outline prospective pathways and methodologies for the future development of FMs in biological research, facilitating ongoing innovation in the field. This comprehensive examination not only serves as an academic reference but also as a roadmap for forthcoming explorations and applications of FMs in biology.

## Introduction

**Figure 1:**
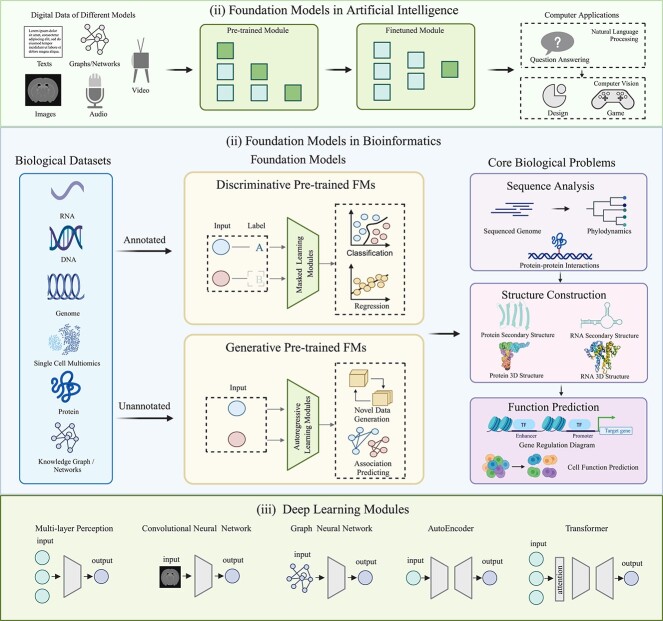
FMs in artificial intelligence (AI) and bioinformatics. (i) FMs in AI. General FMs are predominantly pretrained on diverse digital data and fine-tuned for various computer applications, such as question-answering systems, image design, and computer games. (ii) FMs in bioinformatics. FMs in bioinformatics primarily focus on core biological problems including biological sequence analysis, biological structure construction, and biological function prediction, encompassing both annotated and unannotated biological datasets. They can undergo pretraining on multiple phases of biological data for diverse downstream tasks. Based on the pretraining architectures of the foundation model, they can be classified into discriminative FMs, which capture complex patterns and relationships within annotated data through masking strategies for classification or regression tasks, and generative FMs, which focus on generating semantic features and context from novel data or predicting their associations. (iii) Deep learning modules. Deep learning modules are the cornerstone of building encoders and decoders in FMs. Commonly used modules include MLP, CNN, AutoEncoder (input is consistent with output), GCN (input with graph structure), and transformer (rectangle represents attention mechanism). All these deep learning modules can be trained in an end-to-end manner, enhancing computational efficiency through parallel processing mechanisms.

Bioinformatics endeavors to extract meaningful insights from diverse biological data sources such as amino acid sequences, protein structures, single-cell transcriptomics, biomedical text, and images. These efforts underpin critical applications including disease detection, drug design, and novel therapy discovery. However, their applicability often requires extensive customization for specific datasets over decades [1, 2]. Artificial intelligence (AI), powered by increasing data availability and computational resources, presents an alternative avenue for elucidating biological phenomena through the integration of deep learning mechanisms [3] such as multilayer perceptron (MLP) for nonlinear features [4], convolutional neural network (CNN) for image features [5], recurrent neural network for time series features [6], transformer for natural language features [7], graph neural network (GNN) for graph-represented features [8], and graph attention network for targeted graph features with distinct attention [9]. Foundation models (FMs) are inherently versatile, pretrained on a wide range of data to cater to multiple downstream tasks without requiring reinitialization of parameters. This broad pretraining, focusing on universal learning goals rather than task-specific ones, ensures adaptability in fine-tuning, few-shot, or zero-shot scenarios, significantly enhancing performance and garnering growing attention and popularity within the AI community [10]. General FMs undergo pretraining on digital data and are subsequently fine-tuned for specific computer applications of interest [Fig. 1 (i)]. They have emerged as the state-of-the-art approach for question answering [11], video games [12], AI education [13], medical AI [14], and other applications in computer science.

Recently, FMs have deciphered immense potential in bioinformatics. Compared with large language models (LLMs) that extract meaningful insights from biological texts with natural language processing (NLP) techniques, FMs provide essential groundwork for domain-specific applications, building blocks for specialized LLMs. It tailors LLMs for biological problems by fine-tuning process, narrowing the model representation gap between the general domain and the biological domain. A key strength of FMs lies in their capacity to learn accurate representations of intricate biological datasets. This is facilitated by data-intensive pretraining, a process that researchers can easily utilize for various downstream tasks with limited data through fine-tuning mechanisms (e.g. transfer learning for varying scale biological targets on a pretrained FM). This flexibility allows researchers to employ pretrained embeddings acquired from others to solve their targeting problems. In terms of reproducibility, discriminative FMs offer more robust generalizations in the translation of experimental inputs to outputs. Their flexibility and robustness surpass conventional methods, providing researchers with advantages in deciphering complex biological systems. Moreover, generative FMs can significantly improve biological performance by deeply probing a plethora of underutilized resources that are either unannotated or laden with noise [15]. The robust and reliable nature, along with the strong exploration and exploitation capacities and flexible adaptability to diverse downstream tasks, make FMs a compelling approach for addressing various biological challenges. These challenges include predicting unknown 3D structures or function annotations from sequences [16], discovering rare disease from limited data [17], and analyzing data affected by noise interference or overlap in bioinformatics.

Despite the widespread adoption of FMs across various research fields and industries, there remains a significant gap in a thorough comprehension of FMs in bioinformatics. Previous approaches have often relied on general domains of text/image/video/graph to analyze targets with the help of NLP, image processing, or graph learning methods [18]. However, these methods often fall short of meeting the needs of biological researchers. Typically, there exist at least three critical challenges that hindered the effective application of FMs in addressing biological problems. Firstly, general FMs, designed primarily for computer applications without biological expertise requirements, frequently suffer from model overfitting in bioinformatics. Secondly, most FMs heavily depend on versatile large-scale training datasets, which may not be applicable to domain-specific biological data [19]. As depicted by the futility theorem, predicted results of binding sites may not necessarily translate to functional outcomes *in vivo*, despite their high binding likelihood to *in vitro* sequences [20]. In many cases, solving a specific biological problem requires incorporating specialized insights derived from biological domain knowledge. Finally, the nonlinear deep features extracted from FMs in AI for biological tasks may face challenges regarding biological interpretability and model reliability due to their complex structures and the diverse nature of biological targets.

**Figure 2:**
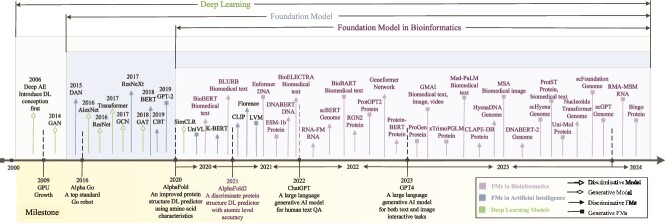
Timeline of FMs in bioinformatics and their background in deep learning. The emergence of FMs in bioinformatics coincided with the ascent of deep learning, gaining significant momentum as these models showcased remarkable advancements in the era of big data. Landmark achievements such as Alpha Go, the first robot to meet top standards, significantly enriched the landscape of deep learning. Subsequent developments, exemplified by AlphaFold and AlphaFold2, revolutionized protein structure prediction from biological sequences. The introduction of GPT4 marked a pivotal moment, catalyzing a surge in the application of FMs. These strides propelled FMs (including discriminative FMs and generative FMs) in bioinformatics to acquire salient information for practical applications in biology.

In this context, a review that encapsulates the state-of-the-art applications of FMs in bioinformatics is valuable and necessary to bridge the existing gap in a comprehensive understanding of these models, shedding light on their operational mechanisms, the contributions they have made, and the challenges and opportunities they present for future endeavors. This review specifically focuses on macromolecules rather than chemical micromolecules, addressing core biological problems such as sequence analysis, structure construction, and function prediction. In response to these challenges, we provide an extensive survey of FMs in bioinformatics. These models, trained in either a discriminative or generative manner, exhibit versatility in their applicability to downstream tasks, including biological multimedia analysis, core biological problems, scMultiomics analysis, and the integration of multimodal biological data. These challenges are intricately tied to various types of biological data, including DNA, RNA, proteins, and single-cell multi-omics data, as well as knowledge graphs/networks, and text/image data, as depicted in Table 1. Further, the evolutionary trajectory of FMs in bioinformatics, as presented in Fig. 2, underscores the development of network structures within deep learning, general FMs in AI, and FMs in bioinformatics with remarkable milestones.

**Table 1:** Biological problems and their associated data in biological FMs. This table provides an overview of five distinct problems in bioinformatics addressed by biological FMs: multimedia analysis, core biological problems (sequence analysis, structure construction, function prediction), single-cell multi-omics (scMultiomics) analysis, and multimodal integration. These problems involve one or more categories of biological data, including DNA, RNA, proteins, scMultiomics, knowledge graphs/networks, and biological text/images. Biological multimedia analysis primarily focuses on biological text/image or video data. Core biological problems involve genes and mutations, biological phenomena data, and their relations and interactions. Multimodal integration biological problems may utilize multiple data types, such as biomedical text/images and proteins.

Problems/Data	Multimedia analysis	Sequence analysis	Construction	Function prediction	Multimodal integration
DNA		*√*		*√*	
RNA			*√*	*√*	
Protein		*√*	*√*	*√*	*√*
scMultiomics		*√*	*√*	*√*	*√*
KGs/Net.		*√*		*√*	
Text/Image	*√*			*√*	*√*

This review delivers an in-depth exploration of recent advancements and challenges related to FMs, with a focus on cultivating a comprehensive understanding of biological issues. While the primary shift involves transitioning FMs from general domains to specialized biological multimedia domains, the review primarily concentrates on three core biological problems critical for analyzing the sequence, structure, and function of biological targets. Additionally, it highlights the significance of analyzing single-cell multi-omics and addressing multimodal integration biological problems, which involve the integration of multiple types of biological data to enhance performance further. The review concludes by deliberating on potential directions in light of current challenges and opportunities. In summary, the exploration of recent FMs in bioinformatics is structured across the following subsections: (i) FM architectures; (ii) biological FMs tailored for five types of biological problems, including the introduction of distinct problems and datasets, data preprocessing, and downstream tasks; (iii) challenges and opportunities; and (iv) conclusions.

## Foundation model architectures

FMs has been primordially observed in NLP [21] and subsequently permeated into computer vision and various other domains of deep learning [22]. In bioinformatics, FMs trained on massive biological data offer unparalleled predictive capabilities through fine-tuning mechanisms. Based on pretraining modules, FMs in bioinformatics can be divided into two main categories: discriminative FMs are primarily designed to capture the semantic or biological meaning of entire sequences by constructing encoders that extract intricate patterns and relationships within annotated data through masked language modeling, resulting in meaningful embeddings. These models excel at tasks like classification and regression, where accurate predictions are derived from well-structured inputs. On the other hand, generative FMs focus on autoregressive methods to generate semantic features and contextual information from unannotated data. These models produce rich representations that are valuable for various downstream applications, particularly in generation tasks where the model must synthesize new data based on learned patterns. The complementary strengths of both discriminative and generative FMs highlight their versatility across a wide array of applications, from precise predictive modeling to creative content generation.

### Discriminative pretrained foundation models

Conventional AI models are typically designed to train neural networks for specific tasks in an end-to-end manner, focusing on optimizing the model for a single task at a time. However, this approach often lacks generalizability and requires significant retraining when applied to new tasks. The advancements in NLP and word embeddings introduced a significant shift in this paradigm, exemplified by BERT (Bidirectional Encoder Representations from Transformers), which marked a breakthrough in embedding capabilities. BERT demonstrated substantial improvements in tasks like summarization, semantic understanding, and sentence classification, underscoring its ability to capture the overall semantic meaning of sequences.

As a discriminative model, BERT [23] leverages variations of masked language modeling during pretraining, where a portion of tokens is masked, and the model is trained to predict these masked tokens. The corresponding loss function is typically cross-entropy, applied to the masked tokens. This bidirectional context allows embeddings to fully capture the semantic nuances of a sequence. Discriminative pre-trained foundation models are particularly effective for classification and regression tasks, enabling a deeper understanding of complex biological processes in both normal and pathological states. These models are typically structured with encoder-only deep learning architectures that employ masking strategies on labeled inputs—such as words and characters—to extract relevant features. These features are then processed through self-attention mechanisms to effectively capture and interpret intricate relationships within the data, enhancing model performance across various applications.

Building on BERT’s success, discriminative pretrained FMs such as BERT have been adapted for specialized domains. For example, in the biomedical field, models like BioBERT [24], BLURB [25], and DNABERT [26] extend BERT’s pipeline to pretrain encoders specifically on biomedical text corpora. These models are designed to capture correlations and associations within large-scale biomedical data, supporting a wide range of downstream tasks such as entity recognition, relation extraction (RE), document classification, and question answering. ProteinBERT [27], for instance, introduces innovative strategies by replacing tokens and introducing false annotations during pretraining, compelling the model to accurately predict the correct sequence information even under challenging conditions. These advancements illustrate how discriminative pretrained FMs continue to evolve, offering robust solutions for increasingly complex and domain-specific applications.

### Generative pretrained foundation models

Generative pretrained foundation models focus on generating coherent sequences by modeling the underlying distribution of the data. Unlike discriminative models, which primarily concentrate on understanding and classifying inputs, generative models are designed to predict the next token in a sequence, making them suitable for tasks that involve generating new content, such as text completion, translation, and content creation. These models are typically trained using autoregressive techniques, where each token is predicted based on previously generated tokens. A well-known example is GPT (Generative Pretrained Transformer), which generates text by predicting each word one at a time, conditioned on all the previous words. In the context of bioinformatics, generative models can be fine-tuned to produce meaningful sequences, such as protein or RNA structures, based on a given prompt. By learning from vast unannotated datasets, these models can generate outputs that are contextually rich and informative for various downstream applications. Generative pretrained FMs excel at capturing complex relationships within data, making them versatile tools for a wide array of tasks beyond classification, including synthesis, creativity, and exploratory data generation. Generative pretrained FMs usually tailor decoder-only modules, such as GPT-3 [28], and GPT-4 V [29], or encoder–decoder, such as ESM-2 [30], ESM3 [31], and T5 models [32], and are adept at simultaneously understanding and generating tasks. These tasks are achieved by optimizing an objective of bidirectional input autoregressive blank-filling, which involves a permuted language modeling approach [33]. For instance, the targeted encoder–decoder model, Enformer [34], predicts promoter–enhancer interactions directly from DNA sequences by leveraging substantial information flow within the CNN. ProtST [35] integrates mask prediction and representation alignment into the pretraining task, facilitating the modeling of paired protein sequences and textual descriptions. ESM3 demonstrates the potential of transformers to generate protein sequences and structures with new functions by training on data produced by evolution. As for the decoder-only model, ProtGPT2 [36] generates protein sequences that exhibit amino acid and disorder properties comparable to those found in natural proteins yet remain distinct from the existing protein space. Additionally, xTrimoPGLM [37] employs four distinct masking strategies to redesign the sequence of complementarity determining region 3 (CDR3).

As shown in Fig. 1 (iii), FMs consist of various neural network modules. MLP with multiple hidden layers in a feedforward neural network is suitable for regression and classification tasks. CNNs, like ResNet, excel in processing grid-like data for vision tasks. The graph convolutional network (GCN) handles graph-structured data, such as molecular graphs, by aggregating information from neighbors. AutoEncoder reduces data dimensions for their representations using encoder and decoder architectures. Transformer, initially proposed for NLP tasks, has positional encodings, self-attention, and multihead attention mechanisms. Generative pretrained FMs leverage these diverse modules for specific biological tasks. For example, the encoder–decoder model Bingo uses a transformer and the GNN for protein function prediction. Decoder-only models such as ProGen, xTrimoPGLM, and CLAPE-DB [38] combine transformer and the CNN for protein sequence, structure, and function analysis. In contrast, encoder-only models, e.g. BioBERT and HyenaDNA, use either transformer or MLP paired with the CNN to address biological text and DNA analysis tasks.

Remarkably, the choice of pretraining strategies holds significant importance in attaining optimal performance [39]. For example, CLAPE-DB combines a pretrained protein language model and constructive learning to predict DNA binding residues [40]. Similarly, HyenaDNA uses a sequence length scheduling technique to stabilize model pretraining and leverages longer context to better adapt to novel tasks [41]. Both discriminative and generative pretrained FMs possess the capability to update and pretrain neural networks via a back-propagation pipeline, leveraging the statistical outcomes of target variables and their estimated counterparts.

### Tuning with foundation models

FMs, particularly in the context of LLMs and biological applications, provide several strategies for interaction depending on the specific needs of a task. These strategies range from zero-shot approaches requiring no additional training to more sophisticated methods that involve fine-tuning or conditional training [24, 25, 35, 41]. Understanding these mechanisms is key to maximizing the effectiveness of FMs across various applications, including bioinformatics.

### Zero-shot learning (no-tuning)

Zero-shot learning enables FMs to perform tasks without any additional training or fine-tuning [25, 42]. In this approach, the model leverages the knowledge it has acquired during pretraining to handle entirely new tasks by relying solely on prompts or queries provided by the user. For instance, in a biological FM like Geneformer [17], BioBERT [24], or scGPT [42], zero-shot learning might be applied to infer the function of a protein sequence based solely on pre-existing general knowledge encoded in the model. This approach is particularly useful when there are limited annotated data available for fine-tuning, as it capitalizes on the model’s broad understanding of biological concepts.

### Few-shot fine-tuning

Few-shot fine-tuning involves providing the model with a small number of labeled examples specific to a task [26, 27]. The model then adapts to the task through minimal additional training. In biological contexts, few-shot fine-tuning is valuable when a model needs to be specialized for niche applications, such as identifying rare genetic mutations or predicting highly specific protein–protein interactions (PPIs). Biological FMs like GMAI [14], BLURB [25], and Enformer [34] can be fine-tuned with just a few examples, making this approach both efficient and effective for tasks where obtaining large datasets is challenging.

### Conditional training and prompt engineering

Conditional training involves adapting foundation models using carefully designed prompts or instructions that guide the model’s output based on task-specific conditions [35, 41]. In the realm of large language models and bioinformatics, this could mean designing prompts that instruct the model to generate hypotheses about molecular structures or to predict biological interactions based on a given sequence. Biological FMs such as ProtST [35], HyenaDNA [41], and ProGen [43] can be used in this approach, often combined with few-shot learning, to allow for more controlled outputs that are aligned with the desired application. Prompt engineering and conditional training are particularly effective when nuanced control over the model’s response is required.

### Foundation models for biological problems

To implement FMs in bioinformatics appropriately, biological problems and datasets together with relevant data preprocessing and downstream tasks in biology will be elucidated at first. As this review concentrates on biological macromolecules (including DNA, RNA, and protein), single-cell genomics, knowledge graphs/networks, text/images, and FMs illustrated in this part are generally employed to solve problems in macromolecule biology. We introduce FMs as versatile tools capable of addressing practical biological problems, including multimedia, sequence analysis, structure construction, function prediction, single-cell multi-omics analysis, and multimodal integration. Each of these areas represents a unique challenge within the field of bioinformatics, and the application of FMs provides innovative approaches to these complex issues. Recent FMs in bioinformatics are summarized in Tables 2 and 3.

**Table 2:** A summary of foundation models in bioinformatics. This table summarizes the model categories, targets, deep module types, and technical advancement of FMs for tackling biological problems (MA, multimedia analysis; SA, sequence analysis; SC, structure construction; FP, function prediction; MI, multimodal integration). FMs are categorized by their pretraining process: DM, discriminative model; GM, generative model. Target biological data types include DNA, RNA, protein, scMultiomics, biomedical text/image/video, and knowledge graph/network. Various deep modules enhance the performance or interpretability of FMs, such as MLP, multilayer perceptron; CNN, convolutional neural network; GNN, graph neural network, and transformer.

Model name	Biological problem	Model category	Targets	Deep module type	Technical advancement	Author name, publication year
BioBERT	MA	DM	Biomedical text	Transformer	Adapt for biomedical corpora by pretrained BERT on large-scale biomedical corpora	Lee *et al.*, 2020
BioELECTRA	MA	DM	Biomedical text	Transformer	A biomedical domain-specific language moBMAl introducing a replaced token prediction pretraining task with generator and discriminator network	Kanakarajan *et al.*, 2021
BLURB	MA	DM	Biomedical text	Transformer	Pretrain biomedical language model from scratch for a wide range of biomedical NLP tasks instead of using complex tagging schemes	Gu *et al.*, 2021
BioBART	MA	GM	Biomedical text	Transformer	A bidirectional and auto-regressive generative language model for biomedical natural language generation tasks along with corresponding data	Yuan *et al*., 2022
Med-PaLM	MA	GM	Biomedical text	Transformer	Introduce HealthSearchQA dataset, propose a human evaluation framework, and present instruction prompt tuning for aligning LLMs to new domains using a few exemplars	Karan *et al.*, 2023
MSA	MA	GM	Biomedical graph	MLP	A medical image segmentation model that fine-tunes the pretrained SAM by integrating the medical-specific domain knowledge	Wu *et al.*, 2023
GMAI	MA	GM	Biomedical text, graph, video	Transformer	Adapt to new tasks due to the acceptance of inputs and production of outputs with varying combinations of data modalities	Moor *et al.*, 2023
DNABERT	SA, FP	DM	DNA	Transformer	Use pretrained bidirectional encoder representation to capture a global and transferrable understanding of genomic DNA sequences	Ji *et al.*, 2021
Enformer	SA	GM	DNA	Transformer	Use a larger receptive field to improve gene expression and promoter–enhancer interaction prediction	Avsec *et al.*, 2021
HyenaDNA	SA, SC	DM	DNA	MLP | CNN	Use a sequence length scheduling technique to stabilize training and leverage longer context length to adapt to novel tasks	Nguyen *et al.*, 2023
Nucleotide Transformer	SA	DM	DNA	Transformer	Build and pretrain foundational language models in genomics, across different genomic datasets and parameter sizes	Dalla-Torre *et al.*, 2023
ProteinBERT	SA, SC	DM	Protein	Transformer	Pretrain protein language model with gene ontology annotation prediction task for both local and global representations	Brandes *et al.*, 2022
ProtGPT2	SA, SC	GM	Protein	Transformer	A generative language model trained on protein space to learn the protein language and produce sequences to sample any region	Ferruz *et al.*, 2022
DNABERT-2	SA, FP	DM	DNA	Transformer	Adapt byte pair encoding to improve computational efficiency and employ multiple strategies to overcome input length constraints	Zhou *et al.*, 2023
ProGen	SA, SC	GM	Protein	CNN | Transformer	A protein language model trained on millions of raw protein sequences that generate artificial proteins across multiple families and functions	Madani *et al.*, 2023
xTrimoPGLM	SA, SC, FP	GM	Protein	CNN | Transformer	A pretraining framework to address protein understanding and generation tasks with joint optimization of the two types of objectives	Chen *et al.*, 2023
CLAPE-DB	SA, SC	DM	Protein	CNN | Transformer	Combines pre-trained protein language model and constructive learning to predict DNA binding residues	Liu *et al.*, 2023
Geneformer	SA, FP	DM	scMultiomics	Transformer	A context-aware, attention-based deep learning model pretrained on a large-scale corpus and can be transferred to diverse fine-tuning tasks	Theodoris *et al*., 2022
scGPT	SA, SC, FP	GM	scMultiomics	Transformer	A single cell foundation model through generative pre-training on over 10 million cells stored by an in-memory data structure	Cui *et al.*, 2023
ESM-1b	SC, FP	GM	Protein	Transformer	Use an unsupervised deep language model to acquire protein structure and function directly from 250 million protein sequences	Rives *et al.*, 2021
AlphaFold2	SC	DM	Protein	Transformer	Improve the AlphaFold by employing an SE(3)-equivariant transformer with an attention mechanism to represent their interactions and distances	Jumper *et al.*, 2021
RGN2	SC	DM	Protein	Transformer	Combine a differentiable recurrent geometric network (RGN) with a transformer-based AminoBERT protein language model to generate backbone structures from unaligned proteins before refinement	Chowdhury *et al*., 2022
Uni-Mol	SC	GM	Protein	Transformer	A 3D position predict model by a 3D molecular pre-training framework along with the candidate protein pre-training for various downstream tasks	Zhou *et al.*, 2023
RNA-FM	SC, FP	DM	RNA	Transformer	Use self-supervised learning to train 23 million non-coding RNA sequences and infer their sequential and evolutionary information	Chen *et al.*, 2022
UNI-RNA	SC, FP	DM	RNA	Transformer	A context-aware foundation model pretrained on an unprecedented scale of RNA sequences unraveling evolutionary and structural information	Wang *et al.*, 2023
RNA-MSM	SC, FP	GM	RNA	Transformer	An RNA language model effective at capturing evolutionary information from homologous sequences using a stack of MSA transformer blocks	Zhang *et al.*, 2024
Bingo	FP	GM	Protein	GNN | Transformer	A large language model and graph neural network (LLM-GNN) based adversarial training method for protein-coding genes prediction	Ma *et al.*, 2024
scFoundation	FP	GM	scMultiomics	Transformer	An extensive single-cell foundation model pre-trained on a dataset of over 50 million single-cell data points with 100 million parameters	Hao *et al.*, 2023
scHyena	FP	GM	scMultiomics	Transformer	A full-length scRNA-seq analysis in the brain by a linear adaptor layer and a bidirectional Hyena operator without losing raw data information	Oh *et al.*, 2023
scBERT	FP	DM	scMultiomics	Transformer	Use self-supervised learning on large-scale unannotated scRNA-seq data to improve the model’s generalizability and overcome the batch effect	Yang *et al.*, 2022
ProtST	FP, MI	GM	Protein, biomedical text	CNN | Transformer	A pretrained framework with three tasks of both protein and biomedical text to boost protein sequence understanding	Xu *et al.*, 2023

**Table 3:** A summary of key characteristics of FMs in bioinformatics.

Model name	Model size	Model task	Model name	Model size	Model task
BioBERT	110 M/340 M	Biomedical text mining (NER, RE, QA)	ProGen	1.2B	Stability prediction, remote homology detection, secondary structure prediction
BioELECTRA	109 M	Biomedical text mining (NER, RE, QA)	ProGen2	6.4B	Functional sequence generation, protein fitness prediction
BLURB	Unknown	Biomedical NLP benchmark (QA, NER, parsing, etc.)	CLAPE-DB	Unknown	Protein–ligand-binding site prediction
BioBART	139 M/400 M	Biomedical text generation (dialogue, summarization, NER)	Geneformer	30 M	Sequence-based prediction
Med-PaLM	12B/84B/562B	Medical question answering	scGPT	Unknown	Multibatch integration, multi-omic integration, cell-type annotation, genetic perturbation prediction, gene network inference
MSA	30 M/100 M	Arabic NLP tasks (NER, POS tagging, sentiment analysis, etc.)	ESM-1b	650 M	Supervised prediction of mutational effect and secondary structure
GMAI	Unknown	Generalist medical AI (multimodal tasks)	AlphaFold2	21 M	Protein structure prediction
DNABERT	110 M	DNA sequence prediction (promoters, TFBSs, splice sites)	AlphaFold3	93 M	Protein structure prediction, structure of protein–protein interaction prediction
Enformer	Unknown	Gene expression prediction	RGN2	110 M	Protein design and analysis of allelic variation or disease mutations
HyenaDNA	7 M	Genomic sequence modeling (regulatory elements, chromatin profiles)	Uni-Mol	1.1B	3D position recovery, masked atom prediction, molecular property prediction
Nucleotide Transformer	500 M ~ 2.5B	DNA sequence analysis	RNA-FM	99.52 M	RNA secondary structure prediction, distance regression task
ProteinBERT	16 M	Bidirectional language modeling of protein sequences, Gene Ontology (GO) annotation prediction	UNI-RNA	25 M/85 M/169 M/400 M	RNA structure and function prediction
ProtGPT	1.6 M/25.2 M	Protein sequence generation	RNA-MSM	Unknown	RNA structure and function prediction
ProtGPT2	738 M	Protein sequence generation, structural similarity detection, stability prediction	Bingo	8 ~ 15 M	Filling in randomly masked amino acids, generating residue-level feature matrix and protein contact map
xTrimoPGLM	100B	Protein understanding and generation	scFoundation	100 M	Gene expression enhancement, tissue drug response prediction, single-cell drug response classification, single-cell perturbation prediction
DNABERT-2	117 M	DNA sequence prediction	scHyena	Unknown	Cell type classification, scRNA-seq imputation
scBERT	Unknown	Single-cell RNA sequencing analysis	ProtST	650 M	Unimodal mask prediction, multimodal representation alignment, multimodal mask prediction

### Biological problems and datasets

FMs can solve practical biological problems that fall within five categories: multimedia analysis, sequence analysis, structure construction, function prediction, single-cell multi-omics analysis, and multimodal integration. Core biological problems are sequence analysis, structure construction, and function prediction. Sequence analysis obtains salient gene information, such as the information of binding sites (commonly encoded as position weight matrices), PPIs, and gene expression, from gene and mutation sequence data DNA, RNA (in which each of four kinds of nucleotides is encoded as a one-hot vector like [1, 0, 0, 0]), protein, and genome (the complex of all the genetic information of an organism). Indeed, the information obtained from these models can be utilized to analyze various downstream tasks. For instance, gene expression embodies the functional regulation process of cells. The differences observed between single-cell genomics pave the way for the discovery of new cell types. Similarly, PPIs and their analogs (such as protein–nucleic acid interactions, protein–ligand interactions, and protein–small molecule interactions) encapsulate the physical binding information between them, providing valuable insights into their interactions. Sequential data are often included as annotations of higher-level data that further contain their synergistic or catalytic interactions.

Structure construction focuses on predicting the structures of proteins and RNA from the secondary structure to the quaternary structure based on the primary structure linear sequences of amino acids in a peptide or protein; the secondary structure contains α-helix, β-sheet with three strands, β-bend, Ω-loop, random coil architecture, and topology targets; the tertiary structure has hydrogen bonds, hydrophobic interactions, and tertiary contacts; and the quaternary structure represents a complex molecule structure. As these structures can be represented by statistical information among amino acid residues [44], many structure construction efforts are made on amino acids in DNA, single-cell genomics, and homologous protein families. Moreover, biological sequence data with different positions may have different functions. In this context, they can also be categorized under multiple sequence alignment (MSA) [16].

Function prediction related to biomedicine enables understanding functions of targets such as proteins and variants to predict polypharmacy side-effects, etc. Core biological data for solving this problem are proteins, individual genes, and their spatial interactions commonly encapsulated within knowledge graphs or networks. These networks represent various information indicated as gene interaction networks, disease pathways, patient networks, and networks that capture similarities between cells. Notably, the prediction of biological function is intrinsically linked to the outcomes of gene expression analysis, given that protein functionality is influenced by the degree of gene expression.

Multimedia analysis involves parsing biological problems by transforming the principles of natural language analysis and computer vision domains into biological areas. Hence, biomedical text like BioBERT [24] and 2D and 3D biomedical images such as microscopy images [19] make up major data in solving domain-specific problems. Multimodal integration biological problems can map multiple types of biological data encompassing multi-omics and morphological histology data, etc.

### Data preprocessing

Data preprocessing is paramount to ensure satisfactory performance before building a model. Original biological datasets may contain multiple inconsistencies caused by varying purposes and acquisition technologies preventing them from being analyzed directly. Adoption of appropriately curated and preprocessed data without exorbitant data overlap, data deficiency, noise interference, or other unexplored data can improve model computational efficiency and representative ability with better model performance. Examples include doublet removal (without duplicate articles), cell-cycle variance removal, data imputation and denoising, dimensionality reduction (reducing the sequence similarity), representation learning, batch effect removal, normalization, background correction, etc.

Doublet removal can avoid mapping duplicate overlapping data with different identifiers or different data that share the same identifying barcode, which plays a significant role in constructing a unique set of relations between entities [45, 46]. Cell-cycle variance removal focuses on removing vain variations in gene expression between cells emerging along the cell cycle by subtracting out the cell cycle influence [47]. It is intractable for data imputation and denoising because only 6%–30% of values can be captured under different chemistry versions and depths, and to decipher “true” and “false” (called “dropout”) zeros in >70% missing values is a guarantee for further identification. To a certain degree, these data can be refined by leveraging similarities with other datasets or through the construction of multiple subneural networks for imputation [48]. Dimensionality reduction of wide gene expression profiles represented in high feature dimensions, also known as representation learning, aims at the construction of embeddings that facilitate the identification of data elements. Systematic variations specific to each batch tend to raise challenges in data integration and lead to significant data wastage. Tran *et al.* compared benchmarks of batch effect removal methods such as seGen (variational autoencoder neural network model and latent space), Scanorama (mutual nearest neighbor and panoramic stitching), and MND-ResNet (residual neural network for calibration) to effectively reduce the variations and batch effects in data captured with different times, types of equipment, or technologies [49]. Therefore, customized fine-tuning can correct sequencing batches from multiple datasets [42]. Protein data can be compared on a common scale by normalization to adjust the measurements [50], and background correction aims to correct for any background noise in the protein data [51].

With preprocessed biological data, the data analysis model can be efficiently employed and mitigate or even eliminate obstacles in biological tasks such as doublet detection and cell-cycle variance annotation. As a result, the judicious utilization of biological data and their corresponding embeddings can significantly enhance the performance of downstream tasks.

### Downstream tasks

In bioinformatics, the analysis of downstream tasks is permitted to evolve through the application of fine-tuning strategies that are desired for accurate performance in analyzing biological problems of interest based on pretrained biological knowledge in FMs. Fine-tuning can greatly reduce computational time and barriers to their implementation and is capable of solving biological tasks related to sequence analysis, structure construction, function prediction, domain exploration, scMultiomics analysis, and multimodal integration.

For sequence analysis, besides traditional sequence alignment analysis [52], homology detection [53], and molecular evolutionary genetics analysis tasks [54], there are promoter interaction prediction, enhancer–promoter interaction prediction, variant identification, variant effect prediction, signal peptide prediction, gene dosage sensitivity predictions, genetic perturbation prediction, protein understanding, DNA replication, stability prediction, etc. Promoter prediction identifies promoter regions of motifs in transcription start sites of genome-wide sequences. Nonpromoter region samples can then be constructed by shuffling and keeping different parts of split promoter sequences with matching lengths. Enhancer–promoter interaction (EPI) prediction is essential in cell differentiation and can interpret noncoding mutation with potential pathogenicity [55]. EPIs are determined by chromatin conformation and thereby can be inferred by chromatin conformation capture-based techniques or other genetic approaches. In addition, the promoters and enhancers are also known as initial and distal regulatory elements, respectively [56].

Variant identification discloses human diseases and traits by distinguishing casual from noncasual variants [34]. Variant effect prediction focuses on determining functional important variants and giving priorities to them [57]. Signal peptide prediction is a binary protein sequence analysis that predicts their presence and locates their cleavage sites [27]. Gene dosage sensitivity predictions present genes that are sensitive to changes in their dosage interpreting copy number variants in genetic diagnosis [17]. Genetic perturbation prediction aims to forecast perturbed original values or perturbed gene expression values in certain tasks [42]. Protein understanding requires accurate representation at the residue level or protein level to understand biological information encoded within proteins [37]. The process of DNA replication is governed by specific initiation and termination sites, with the function of the origin of replication being modulated by epigenetic factors. This intricate process can be studied at a population level by leveraging nontransformed, highly proliferative, and karyotypically stable pluripotent stem cells. Stability prediction calls for statistical representations of protein informatics such as natural language–inspired representations [43].

Structure construction commonly performs secondary or tertiary structure prediction in downstream tasks. Secondary structure prediction was originally achieved by thermodynamic and alignment methods to determine the homologous sequences and their alignments [58]. 3D structures, by contrast, need further exploration due to the lack of 3D structure data, which may be constructed on the raised deep learning method. Moreover, other tasks related to DNA, RNA, protein, and genomics such as predicting DNA binding residues, protein–RNA binding preference, protein–ligand binding pose, splicing junction prediction, neuropeptide cleavage, genome structure and evolution, and gene network, underlie the discovery of their structure information as well. Predicting DNA- and RNA-binding proteins is essential for analyzing genetic variants [59]. Transcription factors (TFs) are binding proteins in regulating gene expression that can bind motifs (specific DNA sequences) to regulate transcription. Generally, pathogenic functional variants in complex neurodegenerative diseases occur with the change of TF-binding intensities [5]. PPI prediction aims at revealing bindings between proteins with transient or stable physical connections. Protein–small molecules and protein–nucleic acid interactions are significant prediction tasks that dominate organism activities [60].

Splicing junction prediction is crucial for protein synthesis and genetic disease identification, whose variant effects can be predicted with the integration of process-specific scores [61]. Neuropeptide cleavage is one of the post-translational modification binary prediction tasks where the maturation of neuropeptides occurs associated with molecule variability for behavioral and physiological states [27]. Genome structure represents genome-regulatory-element secondary structures, and evolution denotes the evolutionary trend of virus variants [58]. Gene network prediction can map networks based on learned connections between genes. Recently, a transfer learning method has been proposed to learn the connection with limited task-specific data showing a promising analysis for rare diseases [17].

Function prediction captures various properties of RNA/protein/gene functions, discoveries (novel) cell type, functional genetic variants, and functional modules and describes gene expression regulation, *in silico* treatment analysis, fitness landscape inference, trajectory inference, etc. Functional properties prediction performs the classification of RNA/protein/gene into several functional groups. For instance, gene function prediction, from classifying gene and protein functions to analyzing genome-wide experimental data with multiple statistical tests, relies on the coverage and accuracy of the annotation data such as Gene Ontology (GO) annotation data [62]. Cell type annotation describes heterogeneity in tissues following cell clustering for further investigation insights into biology and pathology [42]. Functional genetic variants identification probes functional variants located inside regions of interest and subsequently repeats prediction with altered alleles [26]. Functional module detection inputs from networks and functional features to protein complexes and evaluates the overlap of the predicted module and known complex [8].

Gene expression regulation models a biological process where the genetic blueprint within a gene is harnessed to synthesize a functional product. Chromatin state analysis is commonly used for detecting annotation and regulation of the genome and for further nucleosome-level function prediction with gene expression and other related data [63]. Gene expression profile facilitates therapeutic discovery through gene expression similarities measured by distance metric and clinical importance evaluating a certain gene on the gene expression level, e.g. finding a tumor gene compared with normal groups [64]. *in silico* treatment is applied to model human disease by detecting candidate therapeutic targets such as cardiomyopathy and determining the related genes [17]. Fitness landscape inference is developed to map protein fitness under given environments and navigate their residue mutation effect in evolutionary trajectories [35]. Trajectory inference also known as pseudo-time analysis predicts the order or “progress” ranging from the original to the end cell state for single cells from genome-wide omics data [65]. Noticeably, cell ordering, topology, stability, and usability of trajectory inference methods highly depend on the dimension of the dataset and the topology of the trajectory.

Multimedia analysis leverages biomedical text, images, video, etc., for biological domain-specific analysis such as name entity recognition, medical image extraction, medical complementary [24], etc. Prevalent text processing techniques of NLP make numerous efforts to push the progress of mining biomedical text for name entity recognition, RE, sentence similarity, document classification, natural language inference, evidence-based medical information extraction, abstractive summarization, question answering (QA), multiple-choice question answering, etc. Analyzing terms and expressions in the biological domain corpus is pivotal for these tasks. For instance, relation extraction on PubMed enables the discovery of chemical–protein interactions where the majority of relation instances consist of single sentences. In medical vision, they specialize in visual recognition, image captioning, and medical image segmentation. Other domain-specific analyses focus on the medical complementary and alternative, for instance, grounded radiology reports, bedside decision support, augmented procedures, etc.

scMultiomics analysis serves not only single omics downstream tasks such as cell-type annotation, and genetic perturbation prediction but also multi-omics tasks like multi-omics integration relevant to cellular dynamics and disease. Multimodal integration deciphers manifold biological understanding across data modalities such as cross-modal retrieval and multimodal understanding. Besides the aforementioned downstream analysis tasks, many other tasks are not listed or remain to be further studied employing FMs, such as chemical–genetic interaction prediction and other modality-relevant tasks for future biological problems.

### Foundation models for biological multimedia analysis

FMs have extensively explored knowledge within NLP and computer vision [40, 66]. A series of methods such as BERT [23], K-BERT [67], GPT-3 [68], and Dragon [69] utilized FMs to map text, images, knowledge graphs, or their combined data such as Wikidata [70], BoogCorputs [71], and ConceptNet [72], to curate comprehensive language and their complementary domain representation information. Thereby, biological multimedia data, i.e. biomedical text, images, and knowledge graphs/networks built from diagnosis records [73] or other large health datasets [74], could be analyzed in the same way.

Semantic-level biological information encoded within biological text has been a central focus for FMs. Models like BioBERT [24], Med-PaLM [75], BioBLECTRA [76], CodonBERT [77], and BioBART [78] efficiently shift from the general domain to the biological multimedia domain through efficient tokenization. BioBERT identifies a multitude of proper nouns in biomedical texts leveraging its final layer representations to compute token-level BIO2 probabilities exclusively. It also employs sentence classification via a single output layer using BERT’s [CLS] token representation for RE and SQuAD [79] with the same architecture as BERT for the QA task. With minimal architectural modifications, BioBERT accomplishes these tasks by pretraining BERT on large-scale biomedical corpora, such as PubMed [80], resulting in improved performance in biomedical text mining. Med-PaLM combines seven professional medical QA datasets (MMLU clinical topics, LiveQA, MedicationQA, MedQA, MedMCQA, PubMedQA, HealthSearchQA) for aligning the model to new domains using a few exemplars. BioBLECTRA pretrained on PubMed and PMC full-text articles introduces a replaced token prediction pretraining task with a generator and discriminator network. BLURB pretrains a biomedical language model from scratch on unannotated biomedicine text, eliminating the need for complex tagging schemes. Lastly, BioBART, a bidirectional and auto-regressive generative language model designed specifically for biomedical natural language generation tasks, completes the suite with corresponding data.

Besides these biological text-based domain-shift explorations, FMs also incorporate multiple modalities of data. For instance, MSA [81] breakthroughs the lack of training data through a medical-specific domain knowledge–integrated adaptation technique. Similarly, GMAI facilitates easy adaptation to new tasks by accepting inputs with varying combinations of data modalities. With minimal or no task-specific annotated data, GMAI can perform a wide array of tasks, including constructing a comprehensive perspective of a patient’s health status by integrating various modalities, from unstructured symptom descriptions to continuous glucose monitor readings and patient-supplied medication logs.

FMs in bioinformatics exhibit competitive efficacy in explorations tasks, including biomedical text mining (such as named entity recognition, relation extraction, and question answering), PICO (Participants, Interventions, Comparisons and Outcomes entities) extraction, and vision-language extraction. For instance, BioBERT [24], pretrained on biological PubMed abstracts totaling 4.5 billion words and PubMed Central full-text articles totaling 13.5 billion words, outperforms the general domain foundation model BERT [23] Similarly, BioBLECTRA, pretrained from scratch on PubMed abstracts and PubMedBERT, achieves the superior performance of mean test results across all datasets in BLURB.

Model capacity is pivotal to biological multimedia analysis as well. For instance, Med-PaLM, with 540 billion model parameters, achieves a medical QA accuracy of 67.6% on MedQA, surpassing PubMedBERT (38.1%, 100 million parameters) [82], DRAGON (47.5%, 360 million parameters) [69], and PubMed GPT (50.3%, 2.7 billion parameters) [83]. Further, models like MSA, when fine-tuned, achieves the best results compared to SOTA segmentation methods. BioBART also demonstrates competitive performance on biomedical summarization datasets, surpassing BART large by 1.93/1.31/2.1 on Rouge1/2/L on MeQSum. Noticeably, the absence of a standard dataset for training and different training splits could result in lower scores, while the large scale of the model may also present technical obstacles.

### Foundation models for biological sequence analysis

Biological sequence analysis handles exponentially growing sequence data related to genes, mutations, and various biological phenomena. Traditional models typically train identifiers using handcrafted features, necessitating an extra step of manual feature extraction. In contrast, recent works leverage implicit medical knowledge from FMs to tackle specialized tasks and even unseen tasks from unknown sequences. These advancements provide superior prediction results across various tasks with the constraints of correlated biological theory.

Deciphering the language of noncoding DNA to understand how DNA sequence encodes phenotypes represents a major challenge for the next phase of genome biology research. Enformer [34] improves gene expression prediction accuracy, noncoding variant effect prediction, and candidate enhancer prioritization from DNA sequence by integrating long-range interactions in a larger receptive field. Due to the existence of polysemy and distant semantic relationships of noncoding DNA especially in data-scarce scenarios, the gene regulatory code is highly complex. DNABERT [26] pretrains for proximal promoter region identification and fine-tunes two models, DNABERT-Prom-300 and DNABERT-Promscan, using TATA and non-TATA human promoters of 10 000 bp in length from the Eukaryotic Promoter Database (EPDnew). It captures a global and transferrable understanding of DNA sequences after fine-tuning small task-specific annotated data to visualize semantic relationships. When dealing with sequences that extend beyond 512 bp in length, DNABERT segments them into manageable parts and combines their representations to yield the final composite representation. DNABERT-2 [84] further enhances efficiency by incorporating a skilled tokenizer and strategies to handle input length limitations, optimizing time and memory consumption while boosting model capabilities. When extracting semantic-level genome representations, existing processes tend to rely on manual design and generate unsatisfactory representations instead of refined ones that demand costly database explorations. CLAPE-DB [40] leverages pretraining and contrastive learning on vast unannotated data with the ability to handle imbalanced data.

The translation of DNA into proteins, governed by the universal genetic code, relies heavily on the vast information encoded within the genome rather than mere sequential order. HyenaDNA [41] addresses this complexity, leveraging genome sequences across various data lengths and model sizes. Protein sequences across large protein families could be generated through language models. Nucleotide Transformer [57] incorporates information from 3202 diverse human genomes and 850 genomes from a broad spectrum of species, demonstrating that increased diversity improves performance compared to increased model size.

The synthesis of proteins holds immense application potential in areas such as pharmaceutical design and protein engineering. ProGen [43] successfully generates a million artificial sequences after fine-tuning on the curated lysozyme dataset and generates artificial proteins across multiple families and functions. Interactions between proteins and DNA play pivotal roles in vital biological processes such as replication, transcription, and splicing. xTrimoPGLM [37] pretrains a transformer framework with 100 billion parameters to address protein understanding and generation tasks with joint optimization of the two types of objectives. Systematic prioritization obtained from a sequence-based CNN instead of the binary outcome can accurately predict the TF-binding intensities and measure the impact of noncoding variants on TFs. Further, ProteinBERT [27] enables meticulous fine-tuning across an extensive spectrum of protein-related tasks in a remarkably short span of minutes. ProtGPT2 [36] generates sequences with prevalent disorders across datasets displaying 48.64%, 39.70%, and 11.66% alpha-helical, beta-sheet, and coil contents, which is comparable to the natural space with the 45.19%, 41.87%, and 12.93%.

Accurate identification of splice sites is pivotal for ensuring precise protein translation. Among these endeavors, DNABERT outperforms SliceFinder [85] on benchmark data, boasting superior performance with multiclass accuracy of 0.923, an F1 score of 0.919, and an matthews correlation coefficient (MCC) of 0.871 compared to SliceFinder’s reported accuracy of 0.833, an F1 score of 0.828, and an MCC of 0.724. In functional variant prediction, ProGen aligns more accurately with experimentally measured assay data from protein datasets chorismate mutase (CM) and malate dehydrogenase (MDH), boasting an area under the curve (AUC) of 0.94 compared to sequence generation methods from the studies that were specifically designed for these families such as ProteinGan [86] with an AUC of 0.87. HyenaDNA establishes a new state of the art across all datasets. It surpasses previous benchmarks such as GenomicBenchmarks [87] by substantial margins, achieving an improvement of up to 20 percentage points in the task of human enhancer identification.

Protein sequences, akin to natural languages, encapsulate structure and function in their amino acid sequence. ProtGPT datasets exhibit a comparable distribution of ordered and disordered regions across datasets IUPred3 and ordered content. Notably, the proportion of ordered amino acids in ProtGPT2 and natural datasets is 79.71% and 82.59%, respectively, underscoring the similarity in their composition. Specifically, FM xTrimoPGLM achieves a template modeling score (TMscore) of 0.961 in predicting variable heavy (VH) and variable light (VL) structure in antibodies, surpassing AlphaFold2 (0.951) and other advanced methods including OmegaFold [88] (0.946), ESMFold [89] (0.943), IgFold [90] (0.945), and xTrimoAbFold [91] (0.958). While FMs are not anticipated in generating an entirely different distribution or domain, they can expand the variety of sequences sampled by evolution, thereby enhancing model performance.

### Foundation models for biological structure construction

Understanding secondary and 3D biological structures is vital for medical interventions such as vaccine creation, which involves determining the messenger RNA (mRNA) structure. Conventional methods rely on physics-oriented methods like cryogenic electron microscopy, thermodynamic methods supported by experimentally determined thermodynamic parameters, and alignment-oriented methods [85]. The lack of structure datasets and structural instability of genes like RNA have prompted significant efforts in developing computational methods. FMs allow for the creation of a learned, RNA/protein-specific neural network to accurately predict biological structure through token manipulation and position embedding.

FMs offer a scalable combination of data and model capacity for downstream tasks in biological structure construction [92]. For secondary structure prediction, ProteinBERT [27] recovers corrupted inputs by randomly replacing tokens and adding random false annotations, forcing the model to predict annotations from the sequence alone; ProGen [43], trained on millions of raw protein sequences, generates artificial proteins with structural divergence; AlphaFold [44] derives distance maps and torsion distributions between pairs of residues from protein sequences; AlphaFold2 [93] further improves the accuracy using a certain noisy student self-distillation approach and generates a new dataset of predicted structures; ESM-1b [94] trains a deep contextual language model on 86 billion amino acids across 250 million protein sequences; NetSurf [95] replaces the conventional logistic regression linear layer with a deep neural network; xTrimoPGLM [37] pretrains the classification task on helices, strands, and various turns like coils; and CLAPE-DB [40] combines the pretrained protein language model ProtBert [96] with constructive learning to discover a representation space for predicting ligand-binding sites in a protein sequence.

For tertiary structure, FMs can directly predict the positions of biological targets. For instance, Uni-MoI [97] predicts 3D positions by utilizing two pretrained models: a molecular model pre-trained on 209 million molecular conformations and a pocket model pretrained on 3 million candidate protein pocket data. It completes self-supervised pretraining on selected positions with minimal delta positions from random positions, avoiding the need for a masking strategy to recover the correct 3D position [98]. Rapid prediction of protein structure is also indispensable. RGN2 [99] achieves a remarkable reduction in computation time by up to 106-fold, outperforming AlphaFold2 in the analysis of orphan proteins and various classes of designed proteins. Guo *et al.* [100] propose a pretraining model to learn hierarchical structure embeddings from protein tertiary structures to improve prediction efficiency. Moreover, McDermott *et al.* [101] impose relational structure constraints on the pretraining framework and incorporate a pretraining graph as an auxiliary input, whose performance is supported by theoretical results.

FMs in biological structure construction greatly surpass the limits of conventional structure prediction methods. RNA-FM achieves an F1 score of 94.1% and 70.4% on ArchieveII600 and bpRNATS0 datasets, respectively, surpassing SPORT-RNA [102] by 22.8% and 7.5% and notably outperforming the SOTA UFold [103] by 3.4% and 4.0%, respectively. AlphaFold combines bioinformatics and physical approaches to build components from PDB data, enabling the handling of complex physical contexts in challenging cases such as intertwined homomers. As for 3D pose prediction, Uni-Mol predicts 80.35% of binding poses with an root mean square deviation (RMSD) ≤2 Å, better than popular docking methods. When dealing with small-scale data tasks, RNA-FM confirms that the transfer learning employing pretrained parameters of ResNet32 on bpRNA-1 m enables improvement of the task performance by another 20 points compared to a simple ResNet32 with RNA-FM. RNA-FM’s 3D distance prediction attains a pearson product-moment correlation coefficient (PMCC) of 0.8313 when combining sequence encoding, MSA covariances, and RNA-FM embeddings.

The potential of FMs extend beyond structure prediction CLAPE-DB demonstrates superior performance with AUC values of 0.871 and 0.881 on two benchmark datasets, TE46 and TE129 in DNA-binding sites prediction. It outperforms the latest advanced sequence-based models, including DNAPred [104] with AUC values of 0.845 and 0.730, NCBRPred [105] with AUC values of 0.823 and 0.713, and SVMnuc [106] with AUC values of 0.812 and 0.715. With the increasing availability of genomic profiling data and 3D genome contact maps, more types of binding sites can be further identified.

### Foundation models for biological function prediction

Biological functions have garnered significant attention and interest within the domain of bioinformatics. Traditional function prediction models mainly classify targets into one or more categories of collected function datasets such as GO [107] that delineates functions by hierarchical ontologies including molecular functional, biological process, and cellular component [108, 109]. Although GO has >50 000 classes, existent function taxonomy is immature, incomplete, and imbalanced. Further, highly variable genes (HVGs) are mainly selected from the expression variance across the entire dataset. This selection will also remove genes that are stable within the dataset, even though it is crucial to specific cell states in that dataset with respect to all other possible cell states. For instance, in a brain dataset, a neuron-specific transcription factor might be excluded due to its lack of high variability. However, this gene, specific to neurons, is critical for the model’s accurate understanding, distinguishing it from other cell types encountered during pretraining. Here, biological function prediction FMs offer a solution to these challenges.

For instance, Geneformer [17], a context-aware, attention-based deep learning model, leverages pretraining on a corpus of 29.9 million transcriptomes to accurately predict disease genes and their targets. It can be fine-tuned for a variety of downstream tasks related to chromatin and network dynamics. DNABERT [26] provides an accurate prediction of functional genetic variant candidates from ~700 million short variants in dbSNP. xTrimoPGLM [37] employs four distinct masking strategies to redesign the selected sequence and evaluates the implications of synthesized protein sequences associated with specific biological functions. ProtST [35] proposes a multimodal integration pretraining framework on both protein sequences and biomedical texts, outperforming the sequence-based model ESM-1b [95] on protein function annotation. RNA-FM [58] leverages embeddings pretrained on noncoding RNAs to model the function of the 5′ untranslated region in mRNA, showing its versatility in handling noncoding sequences.

Existing methods often require preprocessing of raw data due to their limited capability to model high-dimensional data efficiently. To overcome this challenge, scBERT [110], pretrained on 1 million unannotated single-cell RNA sequencing data, tackles batch effects, increases sequence length, and enhances model generalizability by employing Performer [111], a matrix decomposition transformer. It provides dense embeddings encoded from large-scale unlabeled raw data. To simplify the comparison between pretrained and fine-tuned models, scGPT [42] performs HVG selection as well as a binning technique to model high-dimension data. Specifically, it employs an in-memory data structure tailored for nonsequential omic data, enabling storage of hundreds of datasets and facilitating rapid access to large-scale data. Pretrained on over 10 million cells stored within this in-memory data structure, scGPT converts all expression counts into relative values using a novel value binning technique, partitioning the expression matrix into bins after selecting HVG with *log*1*p* transformation. Similar to creating sentences in natural language that are grammatically and semantically correct on a range of topics, ProtST [35] creates protein sequences with predictable functions across diverse protein families, enabling adaptation to a wide array of protein families. L2P-GNN [112] employs a dual adaptation mechanism at both node and graph levels to encode local and global information, facilitating biological function prediction using 88 000 annotated subgraphs for a 40-binary classification task.

Another limitation of available training data is the imbalance and the presence of extremely similar subtypes. For instance, in the cell type annotation task on the Zheng68k peripheral blood mononuclear cell dataset, the accuracy could not surpass 0.71 with traditional methods. In contrast, FM scBERT acquires an accuracy of 0.759 in the same scenario. Meanwhile, scBERT effectively captures long-range interactions and achieves higher performance on both known and unknown classes. For unknown classes, scBERT achieves an accuracy of 0.329, surpassing SciBet [113] (0.174) and scmap_cluster [114] (0.174), and, for known classes, scBERT achieves an accuracy of 0.942, outperforming SciBet (0.784) and scmap_cluster (0.666). Genomeformer significantly boosts the prediction of central versus peripheral factors (AUC 0.81) compared to other methods (AUC 0.59–0.69). While functional tasks, tied to the distribution of pretrained data, have not yet surpassed structural tasks in terms of improvements, biological function prediction FMs consistently outperform baselines by a large margin and achieve higher accuracy.

### Foundation models for biological scMultiomics analysis

Single-cell multi-omics provides significant insights into cellular dynamics, gene regulation, and disease mechanisms, incorporating complex cellular modalities and states at once [115]. However, integrating diverse data sources often suffers from data scarcity, integration intricacies, and limited robustness and clarity of many modalities such as epigenomics and proteomics, hindering traditional models in maximizing their benefits. FMs bridge this gap by connecting disparate data modalities. For single-cell biology, scGPT [42] integrates multiple sequence data modalities such as DNA, RNA, and protein with a generative pretrained model across a repository of >10 million cells, offering a holistic view of cellular states. It supports joint optimization of multi-omics tokens, i.e. condition-specific tokens, from paired data in the flexible embedding architecture. Compared with scGLUE and Seurate v4, which produce a merged cluster of B cells with three major types, scGPT can differentiate these three types of B cells into distinct groups and further provides a clear separate cluster for CD8 naive cells with a superior performance in overall clustering (AvgBIO = 0.767).

Besides single-cell multi-omics, other multi-omics FMs also hold immense promise, bridging the gap between data modalities and propelling us toward a deeper understanding of cellular dynamics and disease. For instance, scFoundation [116] serves as a versatile FM, harmonizing diverse biological data sources including sequence information, structural features, and chemical compounds. It integrates the cancer cell line encyclopedia (CCLE) and genomics of cancer drug sensitivity (GDSC) datasets for input cell line gene expression, the drugs, and IC50 labels, extracting transcriptome features fed into subsequent prediction modules. scFoundation enables transferring gene relationships to bulk-level expression data, improving IC50 prediction accuracy. As the amount of publicly available multi-omics data continued to expand, future FMs may enable more meaningful predictions in elusive tasks even with limited task-specific data.

### Foundation models for multimodal integration biological problems

Recent language understanding research suggests that the text-corpora pretrained models are surprisingly effective, shedding light on the potential for multimodal analysis [117]. Traditional biological models mainly focus on unimodal information and have difficulties in handling multimodal data or multilevel data [35, 118, 119]. While FMs offer a broader understanding of targets, they are also vulnerable to perturbation and necessitate specific fine-tuning approaches. Multimodal integration enables a deeper understanding of diverse topics for a systematic study of biological samples [58]. For instance, scGPT [42] enables multi-omics prediction through generative AI with the integration of expression and new condition tokens to extend the embedding architecture to accommodate multiple modalities, batches, and states. Similarly, ProtST [35] improves the original representational capacity of the protein language model through the application of multimodal representation alignment and multimodal mask prediction.

It is imperative for biological FMs to incorporate a more diverse range of data types, e.g. temporal data and perturbation data. For instance, analysis of knowledge graphs often suffers from low accuracy due to the data imbalance problem, where richer entities possess more relations and information, but the scarce ones will not be fully represented with limited information. A possible solution is to incorporate varying forms of biological data such as sequence data, structure data, and chemical compounds. Leveraging exponentially growing biological data alongside advanced FMs holds promise for achieving both clinically and biologically significant outcomes.

## Challenges and opportunities

**Figure 3:**
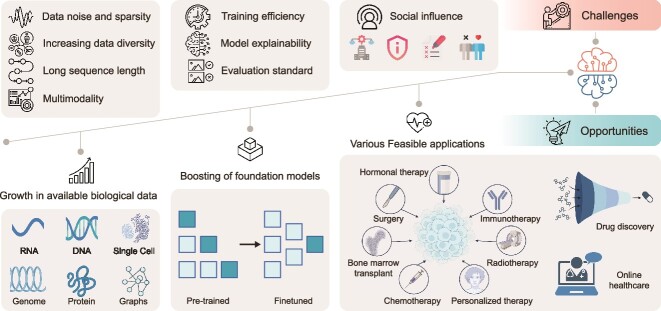
Challenges and opportunities in applying FMs for biological problems. FMs for addressing biological problems face hurdles related to biological data, model structures, and their social influence, which concurrently catalyzes opportunities in bioinformatics due to the increasing availability of biological data, advancements in FMs, and their versatile real-world applications. The top half of this figure outlines challenges such as data noise and sparsity, increasing data diversity, long sequence length, and multimodality in biological data collection. Additionally, challenges in training efficiency, model explainability, and establishing evaluation standards in model design and construction are depicted. Social influences, including ethics and fairness, privacy concerns, potential misuse, and social bias, further compound these challenges. Conversely, the bottom half of the figure illustrates emerging opportunities driven by the proliferation of diverse biological data types and volumes, including RNA, DNA, scMultiomics, proteins, and knowledge graphs/networks. The enhancement of FMs, particularly through pretrained mechanisms, presents another avenue for progress. Moreover, a wide range of applications spanning surgery, hormonal therapy, immunotherapy, radiotherapy, personalized therapy, chemotherapy, bone marrow transplant, drug discovery, and online healthcare, underscore the potential impact of FMs in bioinformatics. These developments signal a promising trajectory for the application of FMs in addressing biological complexities.

FMs are a double-edged sword, presenting both opportunities and challenges [120] [121]. While they make large biological data analysis possible, they also demand substantial computing resources, entail a vast number of model parameters, and suffer from low explainability and reliability. These challenges, along with potential opportunities for advancing promising biological areas, are depicted separately in Fig. 3 and are further elaborated below.

## Challenges

### Data noise, sparsity, and diversity

FMs derive embeddings to support other biological tasks still grapple with the sparse or corrupted noisy data [122, 123]. The sparsity of biological data typically stems from data collection deficiencies, with variations in chemistry versions and depths of data capture, as well as imbalances in research focus that tend to concentrate on well-known phenomena. Noises and biases often manifest in different selection strategies, experimental conditions, and other factors. While some FMs indicate that this issue could be alleviated through careful review of these data and deep investigation of the phenomenon, they remain susceptible to data corruption present in existing evaluation sets or in future data capture scenarios in the real world. Increasing data diversity holds the promise of improving model performance efficiently [95]. However, diversity with task-unrelated data in real-world scenarios may not be readily transferred to downstream tasks [124, 125]. To derive accurate representations from diverse biological data, FMs can emphasize both feature-level and semantic-level training strategies to harmonize biological knowledge across modalities [126].

### Long sequence length

Biological sequence holds great potential for addressing diverse biological challenges, but their extensive length poses significant challenges during model training. Consider a single human, for instance, with ~3 billion nucleotides, or a bacterium with 5 million, or even a virus with 30 000. These lengthy sequences introduce considerable gradient variance, leading to increased instability and reduced training efficiency. Inspired by Sortformer [127], Li *et al.* [128] have endeavored to achieve stable training with reduced computational cost by improving data efficiency. However, the method’s utilization of varying sequence lengths, obtained directly through truncation, entails sacrificing the information from dropped data. Causal relationships, prerequisites, or other significant factors have yet to be adequately represented and validated. This could be addressed by further leveraging data localization, structural and functional aspects, or other chemical and biological rules and relationships.

### Training efficiency

Pretraining constitutes a vital step for most of the FMs to maintain coherence within each shot. However, the high computational costs on huge amounts of data remain a significant barrier to their widespread implementation. For instance, AlphaFold2 requires several weeks of training on up to 200 graphics processing units (GPUs). To improve the efficiency in analyzing big data, previous approaches have employed various strategies. These include leveraging attention mechanisms such as FlashAttention [129] and Multi-Query Attention [130], kernels [131], sparse activation [65], and other advanced mechanisms to reduce both the model’s training time and detection time. Similarly, substantial redundant computations could be cut down in FMs with advanced technologies that focus on removing unimportant parameters, reducing memory consumption, enhancing convergence rate, paralleling data processing, and fully exploiting the generative and adaptive capabilities of models [92]. In summary, further efforts along these lines are essential for continually improving the efficiency of FMs, thereby enhancing their applicability in diverse domains.

### Model explainability

It is also challenging to provide interpretability for FMs in each step and acquisitions with logical evidence in bioinformatics. Clear and robust explainability and interpretability are significant factors of highly comparative prediction accuracy enabling a wide range of biomedical and healthcare applications to explain the model and results to consumers and researchers [132]. Efforts have been made to explain them in biological applications such as scBERT, which elucidates the contribution of genes and their interactions by analyzing attention weights within the self-attention mechanism in the model for gene exploration and decision-making tasks. Thereby, the top genes could be visualized by the weights and analyzed in the following stages [110]. However, this approach relies solely on structural results, which neither indicate the importance of each node nor provide explanations for the model’s reliable results. We envision that FMs can dramatically improve interpretability and explainability by incorporating knowledge graphs and networks to narrow the gap between FMs and experts for solving more complex biological problems.

### Evaluation standard

Traditional AI-based models designed for specific tasks in computer vision or natural language processing are typically evaluated based on predefined metrics, making it straightforward to assess their performance. However, FMs face various downstream tasks as well as unseen tasks, making it uniquely challenging to anticipate all of the modes and set an evaluation standard for these methods. Current qualitative evaluations often focus on certain modules such as Machine Reading Comprehension within a complete QA pipeline, rather than assessing previously unseen tasks, such as diagnosing disease in brain magnetic resonance imaging [14]. Additionally, the general domain evaluation may overlook the impact of rich biological regulations, such as biomedical synonymous relationships [133]. To accurately evaluate model performance and output quality, which, in turn, prevents the occurrence of overly confident assertions, it is essential to consider model uncertainty and incorporate biological knowledge from domains such as radiology, pathology, and oncology.

## Opportunities

### Biological data

The exponential growth of available biological data, such as datasets for RNA secondary structure prediction like bpRNA-1m [134] (102 318 sequences from 2588 RNA families), is expected to significantly enhance the performance of FMs on downstream tasks. Despite the availability of extensive datasets, there remains a vast amount of untapped data that FMs have yet to fully utilize. Complex combinations of biological information or conditional data offer promising avenues for further analysis by FMs.

### Foundation model architecture

Transitioning from a derivable approach to a multifocus framework presents difficulties. In this respect, we can discuss FMs from two perspectives. First, with controllable biological data and model size, designing different strategies for different data and tasks becomes feasible. Second, dealing with particularly large models that contain extensive biological information and a massive number of parameters requires quick and stable learning. Despite feasible efforts, the current cognitive abilities of FMs still fall short of expectations. To this end, developing new training strategies for FMs is of paramount significance.

### Feasible applications

FMs enable various bioinformatics applications, particularly in disease understanding and therapy, drug discovery, and personalized medicine. Regarding the therapy of cancers, FMs could provide physiological function insights into targets and potentially replace traditional analytical methods for building cancer prognosis models [135]. In drug discovery, FMs can uncover corresponding phenomena, while in personalized medicine, they can design drugs based on a patient’s genetics, genome, and health history. Additionally, in online healthcare, FMs serve as a central storage and backbone of healthcare systems, powering question-answering systems and healthcare-assistive robots to leverage medical data and resources effectively [136].

## Conclusions

In conclusion, with the rapid iteration and development of AI, the surge in data availability has brought unexpected opportunities and challenges for the field of bioinformatics. The vast increase in both annotated and unannotated biological data, coupled with AI advancements, provides an ideal environment for leveraging foundation models to transform computational biology. Below, we outline the key contributions of this survey:

- Enhancements in bioinformatics through FMs: This survey illustrates how FMs have significantly advanced bioinformatics by addressing challenges with abundant unannotated data. Through pretraining on large and diverse datasets, FMs have demonstrated a remarkable capacity for capturing intricate biological representations, achieving state-of-the-art results in the analysis of biological sequences, structures, and functions. Their ability to generalize knowledge across various biological contexts makes FMs powerful tools for advancing research in computational biology.
- Comparative strengths and challenges of FMs: We emphasize the advantages of FMs over traditional bioinformatics methods, highlighting their adaptability, superior performance, and ability to represent complex biological information. Unlike conventional models that often require task-specific training, FMs can be flexibly fine-tuned or used in zero-shot settings, making them versatile across a wide range of biological tasks. However, FMs also face notable challenges, including dealing with data sparsity, handling noisy or incomplete data, managing the computational costs of training on long biological sequences, and providing interpretable results in biological applications. Addressing these challenges is critical for further advancing their utility in the field.
- Guidance and future prospects for innovations: This survey offers valuable insights for future research by summarizing the current applications and achievements of FMs in tackling biological challenges. We outline potential pathways for advancing FMs in bioinformatics, emphasizing the integration of domain-specific knowledge to narrow the gap between general AI and biological applications. Moreover, we advocate for the development of advanced training strategies and improved model architectures to enhance interpretability, efficiency, and overall performance. These prospects guide the research community toward overcoming current limitations and unlocking new opportunities for innovation in computational biology.

Conflict of interest: None declared.

## Data Availability

No datasets have been utilized in this review paper.
